# Comprehensive assessment of of thoracic stent grafts after emergency implantation in multi trauma patients

**DOI:** 10.1186/1532-429X-11-S1-P168

**Published:** 2009-01-28

**Authors:** Volker Rasche, Axel Bornstedt, Vinzent Hombach, Nico Merkle, Alexander Oberhuber, Ludger Sunder-Plassmann, Martin Hoffmann

**Affiliations:** University Ulm, Ulm, Germany

**Keywords:** Stent Graft, Endovascular Stent Graft, Endovascular Aortic Repair, Aortic Stent Graft, Thoracic Endovascular Aortic Repair

## Introduction

Thoracic Endovascular Aortic Repair (TEVAR) of various aortic pathologies has turned out as attractive alternative option to conventional surgical approaches. TEVAR may be associated with graft related complications such as endoleaks, kinking, infolding, and stentgraft migration, disconformability and disattachment phenomena. Therefore lifelong regular follow up by tomographic imaging is required for closely monitoring of all patients treated with thoracic aortic stent grafts.

Multislice volumetric CT still represents the imaging gold standard for the assessment of the stent graft. However, its related X-ray dose and the nephrotoxicity of the required contrast agent, limits its frequent application especially in younger patients and in patients with renal insufficiency. The objective of this work was to proof the feasibility of MRI as a comprehensive imaging tool for the assessment of thoracic stent grafts, including disattachment phenomena, its dynamics over the cardiac cycle and its impact on vessel compliance.

## Methods

Twelve consecutive patients (3 female, 9 male; mean age 38 +/- 18; 8 – 2417d after intervention) were enrolled in this feasibility study. All patients initially presented with aortic rupture at the transition zone of the arch and descending segment of the thoracic aorta. The rupture site was treated with endovascular stent graft procedures in all patients. In six patients, a Medtronic Valiant stent graft and in six patients a Gore excluder TAG stent graft were implanted. All patients underwent conventional contrast agent (CA) enhanced routine CTA followed by the investigational MRI protocol, comprising a three-dimensional angiogram of the aorta with and without contrast enhancement.

The general image quality regarding appreciation of the stent graft geometry Q_G_ and dynamics Q_D_ were ranked on a 1 (poor) – 5 (excellent) scale by three experienced MRI readers. Qualitative assessment of the stent graft motion Q_M_ over the cardiac cycle was ranked on a 1 (no motion) to 3 (significant motion) scale independently for the proximal and distal segments of the stent. The deployment of the stent Q_d_ against the vessel wall was qualitatively assessed on a 1 (attached to the vessel wall) to 3 (large areas of the stent not attached to the vessel wall) scale. Quantitative assessment of the vessel compliance was performed distal and proximal to the stent graft as well as in the distal and proximal section of the stent graft.

## Results

**Figure 1:**
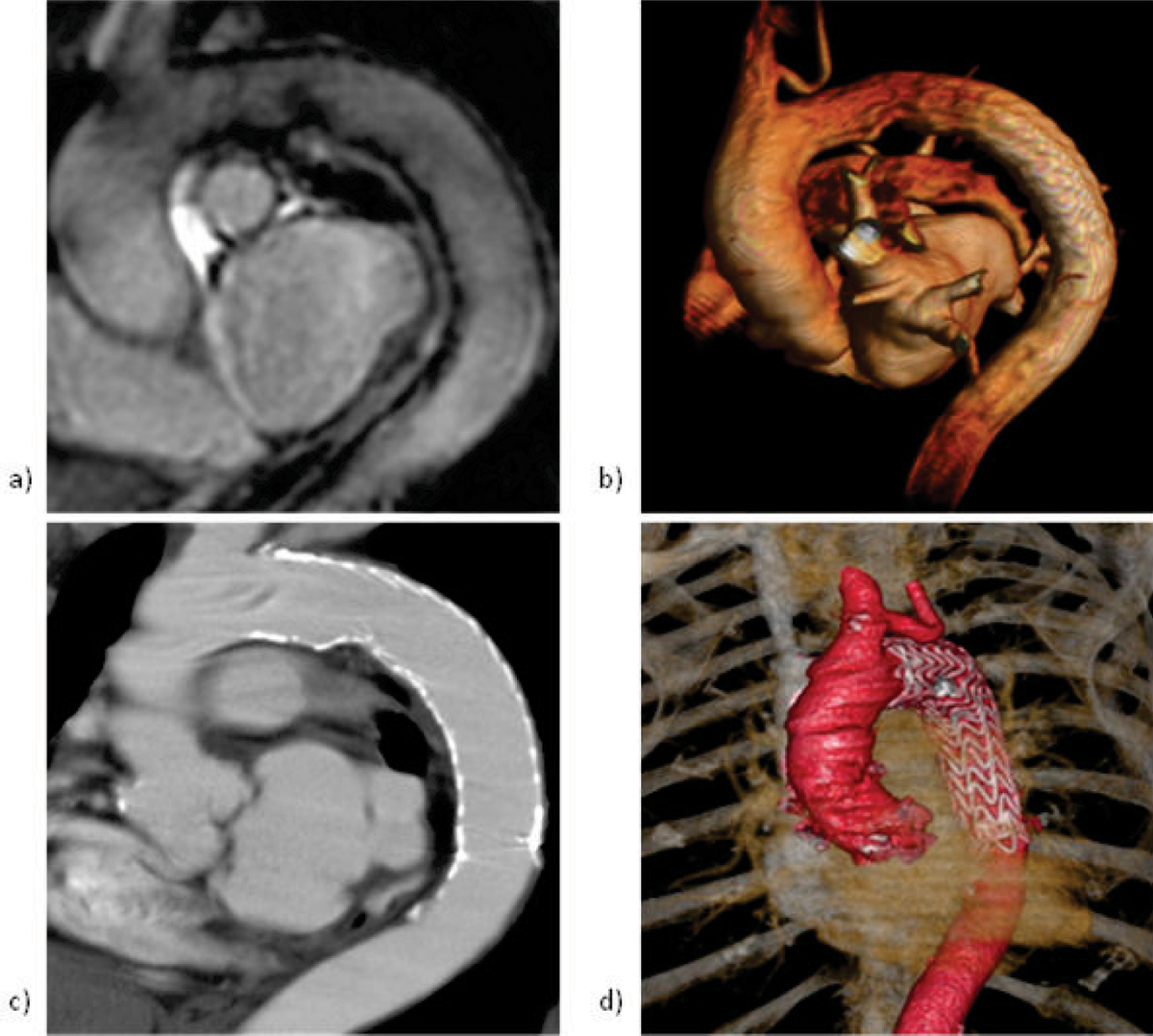
**Comparison MRI and CT: a,c) parasagittal two-dimensional and b,d) volume-rendered view of the aortic arch and the thoracic descending aorta**.

The MRI imaging protocol could be completed in all patients. The average acquisition time for the entire MRI examination was 54 +/- 16 minutes. In all patients, the geometry of the stent graft as well as its dynamic could be obtained with at least mediocre image quality (mean ranking Q_G_ = 4.2 +/- 0.75; mean ranking Q_D_ = 3.7 +/- 0.76). Stent graft motion could be assessed qualitatively and vessel compliances be quantitized in all patients. In direct comparison to CT (Figure 1), no obvious loss of morphological information of the stented thoracic aorta was observed.

## Discussion

MRI can provide a comprehensive assessment of thoracic aortic stents grafts after percutaneous implantation. The feasibility of assessing the stent graft geometry as well as motion without any need for ionizing radiation or nephrotoxic contrast agents, may MRI make a very attractive alternative to the current standard CTA as imaging modality of choice for regular follow up of thoracic stent grafts.

